# CTLA-4 blockade with ipilimumab: biology, safety, efficacy, and future considerations

**DOI:** 10.1002/cam4.371

**Published:** 2015-01-25

**Authors:** Luis H Camacho

**Affiliations:** St. Luke's Cancer CenterHouston, Texas

**Keywords:** Melanoma, CTLA4, CTLA-4, ipilimumab, immunotherapy, survival

## Abstract

Melanoma remains a critical public health problem worldwide. Patients with stage IV disease have very poor prognosis and their 1-year survival rate is only 25%. Until recently, systemic treatments with a positive impact on overall survival (OS) had remained elusive. In recent years, the United States Food and Drug Administration (FDA) – approved several novel agents targeting the RAS/RAF/MEK/ERK pathway (vemurafenib, dabrafenib, and trametinib) – critical in cell division and proliferation of melanoma, and an immune checkpoint inhibitor (ipilimumab) directed against the cytotoxic T lymphocyte Antigen - (CTLA-4). Moreover, recent reports of clinical trials studying other immune checkpoint modulating agents will most likely result in their FDA approval within the next months. This review focuses on ipilimumab, its safety and efficacy, and future considerations. Ipilimumab has demonstrated a positive OS impact after a several-year follow-up. It is also recognized that due to its mechanism of action, the response patterns to ipilimumab can differ from those observed in patients following treatment with conventional cytotoxic agents and even the most recently approved BRAF inhibitors. Most patients (84.8%) experience drug-related adverse events (AEs) of any grade; most of these are mild to moderate and immune mediated. However, a minority of patients may also experience severe and life-threatening AEs. In clinical studies, AEs were managed according to guidelines that emphasized close clinical monitoring and early use of corticosteroids when appropriate. Preliminary results have taught us the potential greater toxicity when in combination with vemurafenib, and the greater antitumor efficacy when combined with nivolumab, a monoclonal antibody directed against programmed death receptor-1 (PD-1), another immune checkpoint inhibitor. Future challenges include the optimization of dosing and toxicities when used as a single agent, and studying the safety and efficacy of combinations with targeted small molecules and other monoclonal antibodies to treat patients with melanoma and other malignancies.

## Melanoma

Melanoma remains a critical public health problem in the United States. It is estimated that 76,100 Americans will be diagnosed with this condition by the end of 2014. The probability of developing invasive melanoma from birth to death has been estimated to be 2.9 (1 in 34) for males, and 1.9 (1 in 53) for females 1. The incidence of melanoma will continue to rise in the United States, at least until the majority of the current population in the middle-age groups becomes the oldest population and adopts better sun exposure habits 2. When diagnosed in its early stages, treatment is intended to be curative. However, for patients diagnosed with unresectable stage III (nodal involvement) or stage IV (distant metastasis) disease, or for those who recur with advanced disease, the associated clinical burden is significant and the prognosis is poor. Historical benchmarks from a recent meta-analysis estimated a 1-year survival rate of only 25% for patients with stage IV disease 3. In this setting, the principal goal of therapy is to extend survival whenever possible.

The last three decades of research has resulted in the approval of four agents with improved survival of patients with unresectable and advanced disease. In 2002, Davies et al. identified BRAF somatic missense mutations in 66% of malignant melanomas and at lower frequency in a wide range of human cancers 4. Research derived from these observations resulted in the approval of three novel directed therapies targeting the RAS/RAF/MEK/ERK pathway: two inhibitors of mutated BRAF tyrosine kinase (vemurafenib and dabrafenib) 5,6, and an inhibitor of mitogen-activated protein kinase (MAPK) kinases 1 and 2 (MEK) (trametinib) 7. Trametinib and dabrafenib are also approved for treatment in combination with each other 8.

### Vemurafenib

Vemurafenib (Zelboraf, Genentech, Inc., South San Francisco, CA) was approved on 17 August 2011, for the treatment of patients with unresectable or metastatic melanoma carrying a *BRAF* V600E mutation. It is estimated that approximately 45% of all melanoma patients bear this mutation in their tumors 9. Vemurafenib has reported interim 6-month phase III data demonstrating improved rates of overall survival (OS) and progression-free survival (PFS) over dacarbazine in 675 patients with previously treated, metastatic melanoma 5. The OS at 6 months was 84% for patients treated with vemurafenib compared with 64% with dacarbazine, whereas the PFS for 549 evaluable patients was 5.3 months with vemurafenib compared with 1.6 months with dacarbazine.

### Dabrafenib

Dabrafenib (Tafinlar; GlaxoSmithKline, LLC, Research Triangle Park, NC), was approved on 29 May 2013, for the treatment of patients with unresectable or metastatic melanoma with BRAFV600E mutation 6. Subsequently, on 10 January 2014, the FDA granted its accelerated approval in combination with trametinib (Mekinist; GlaxoSmithKline, LLC) for use in combination to treat patients with unresectable or metastatic melanoma with a BRAFV600E or V600K mutation 7,8.

Single-agent dabrafenib was approved on the basis of improved PFS in a multicenter open-label randomized (3:1), active-controlled trial. The study screened 733 patients and enrolled 250 of them with previously untreated, unresectable stage III or stage IV BRAFV600E mutation-positive melanoma. Patients who received dabrafenib experienced a statistically significant improvement in the PFS compared with those treated with dacarbazine (HR 0.33; *P *<* *0.0001). The median PFS was 5.1 months for patients treated with dabrafenib and 2.7 months for patients treated with dacarbazine. The objective response rate (ORR) was 52% for patients treated with dabrafenib and 17% for patients treated with dacarbazine. The median duration of response was approximately 5 months for both treatment groups. OS was not statistically different among the groups.

### Trametinib

Single-agent trametinib was approved for the treatment of patients with BRAFV600E or V600K mutation-positive unresectable or metastatic melanoma on 29 May 2013, on the basis of improved PFS in a multicenter international open-label randomized (2:1), active-controlled trial that enrolled 322 patients with BRAFV600E or V600K mutation-positive stage IIIc or IV melanoma. Patients received trametinib (2 mg) once daily or IV dacarbazine (1000 mg/m^2^) or paclitaxel (175 mg/m^2^) every 3 weeks. Cross-over from chemotherapy to trametinib was allowed. The median PFS in the trametinib group was greater than in patients treated with chemotherapy (4.8 months vs. 1.5 months; *P *<* *0.001). Interestingly, in contrast with an incidence of cutaneous squamous cell carcinoma of approximately 20% during therapy with vemurafenib 5, this study did not observe secondary cutaneous neoplasms with trametinib 7.

The combination therapy with trametinib (Mekinist tablets; GlaxoSmithKline, LLC) and dabrafenib (Tafinlar capsules; GlaxoSmithKline, LLC) for patients with unresectable or metastatic BRAFV600E or V600K mutation-positive melanoma was approved on 10 January 2014. This approval was based on durable objective responses confirmed in a multicenter, open-label, randomized, active-controlled, dose-ranging clinical trial that enrolled 162 patients with stage IIIC or IV BRAFV600E or V600K mutation-positive melanoma 8.

## CTLA-4 as a Therapeutic Target

In 1987, Brunet et al. described cytotoxic T lymphocyte antigen-4 (CTLA-4), a 223–amino-acid protein belonging to the immunoglobulin superfamily mainly expressed in activated lymphocytes and coinduced with T-cell–mediated cytotoxicity 10. The human homolog of the gene was subsequently cloned in 1988 11. Shortly thereafter, Krummel et al. described the opposing effects of CTLA-4 and CD28 while attempting to elucidate the processes involved in T-cell activation 12.

Following the work by Allison's group, the development of agents targeting this mechanism commenced. Initially, three antibodies entered clinical trials: tremelimumab (CP-642,206, ticilimumab; Pfizer, Inc., New London, CT), its parental antibody known as CP-642,570 (discontinued because of treatment-related thrombocytopenia in its first-in-human study), and MDX-010 (Bristol-Myers Squibb/Medarex, Princeton, NJ), later renamed to ipilimumab. Tremelimumab demonstrated predictable safety and efficacy in phase I 13 and phase II trials 14, but ultimately failed to meet its end points in phase III 15. This antibody resumed its development by MedImmune/AstraZeneca since 2011 (MedImmune, Gaithersburg, MD; AstraZeneca, London, U.K.). In the same year, ipilimumab received FDA approval for the treatment of unresectable stage III and metastatic melanoma 16. Due to the extensive experience with ipilimumab, this article will focus mainly on this compound, and discuss future directions in its development.

### Mechanism of action

T-cell activation requires two sequential signals 17–21. In a first step, antigens presented in context with the major histocompatibility complex (MHC) I or II on specialized antigen-presenting cells (APCs) bind with T-cell receptors (TCRs). The second step involves translation of TCR stimulation into T-cell activation and requires a costimulatory signal, achieved when B7 molecules on the APC surface bind with CD28 receptors on the T-cell surface. Subsequently, T-cell surface expression of an inhibitory molecule, CTLA-4, takes place. CTLA-4 competitively inhibits the binding of B7 to CD28 by interacting with the same ligands and prevents the costimulatory signal, dampening T-cell activation and proliferation.

Ipilimumab is an IgG1 fully human monoclonal antibody that inhibits CTLA-4 leading to enhanced T-cell activation. After initial preclinical studies that supported proof of concept demonstrating that antibodies directed against CTLA-4 could induce tumor regression, ipilimumab was developed clinically. The most extensive clinical development has been in advanced melanoma 18.

## Ipilimumab Long-Term Efficacy

For decades, agents or combination regimens in development for treatment of advanced or metastatic melanoma were, at best, able to demonstrate increased response rates or PFS but failed to improve OS 22. Ipilimumab was the first immunotherapy to demonstrate improvement in OS in this patient population with high unmet medical needs, changing the therapeutic landscape for this disease in early 2011. In two phase III trials in both the first- and second-line settings, patients with metastatic melanoma achieved long-term, durable responses and improved OS 16,23. In the registration trial reported by Hodi et al. 676 patients with previously treated metastatic melanoma were randomized to receive either 3 mg/kg of ipilimumab plus placebo, ipilimumab in combination with the experimental peptide vaccine gp100, or gp100 plus placebo (Table1 16,23–27). Of note, patients with characteristics that are associated with particularly poor survival, such as high serum lactic dehydrogenase, metastases to the brain, or M1c disease, were included in the trial. The median OS was significantly greater with ipilimumab plus gp100 than with gp100 alone (10.0 months vs. 6.4 months; HR: 0.68; *P *<* *0.001). Median OS was also significantly greater with ipilimumab alone than with gp100 alone (10.1 months vs. 6.4 months; HR: 0.66; *P *=* *0.003). One- and 2-year OS rates for ipilimumab alone were 45.6% and 23.5%, respectively; for gp100 alone, 25.3% and 13.7%, respectively; and for ipilimumab plus gp100, 43.6% and 21.6%, respectively.

**Table 1:** OS rates with ipilimumab in phase II and III studies 16,23–27

Study	Survival rate, % (95% CI)^1^			
	1-year	2-year	3-year	4-year
Hodi et al. 16,2 (*N *=* *676)				
3 mg/kg + gp100, previously treated (*N *=* *403)	43.6	21.6	N/R	N/R
3 mg/kg, previously treated (*N *=* *137)	45.6	23.5	N/R	N/R
Gp100, previously treated (*N *=* *136)	25.3	13.7	N/R	N/R
Robert et al. 23,2 (*N *=* *502)				
10 mg/kg + DTIC, treatment naïve (*N *=* *250)	47.3	28.5	20.8	N/R
DTIC, treatment naïve (*N *=* *252)	36.3	17.9	12.2	N/R
O'Day et al. 24,25 (*N *=* *155)				
10 mg/kg, previously treated	47.2 (39.5–55.1)	32.8 (25.4–40.5)	23.3 (16.7–30.4)	19.7 (13.4–26.5)
Wolchok et al. 25,26 (*N *=* *217)				
10 mg/kg, previously treated *(n *=* *72)	48.6 (36.8–60.4)	29.8 (19.1–41.1)	24.8 (14.8–35.7)	21.5 (11.9–32.0)
3 mg/kg, previously treated (*n *=* *72)^3^	39.3 (28.0–50.9)	24.2 (14.4–34.8)	19.7 (10.7–29.4)	18.2 (9.5–27.6)
0.3 mg/kg, previously treated (*n *=* *73)^3^	39.6 (28.2–51.2)	18.4 (9.6–28.2)	13.8 (6.1–22.5)	13.8 (6.1–22.5)
Weber et al. 25,27 (*N *=* *115)				
Ipilimumab + placebo (*N *=* *57)	62.4 (49.4–75.1)	41.8 (28.3–55.5)	34.4 (21.1–48.2)	32.0 (18.9–45.7)
10 mg/kg, treatment naïve (*N *=* *32)	71.4 (55.2–87.2)	56.6 (38.4–74.3)	42.5 (23.0–62.0)	37.7 (18.6–57.4)
10 mg/kg, previously treated (*N *=* *25)	50.8 (31.5–71.1)	24.2 (8.0–42.8)	24.2 (8.0–42.8)	24.2 (8.0–42.8)
Ipilimumab + budesonide (*N *=* *58)	55.9 (42.7–68.8)	41.1 (27.7–54.8)	38.7 (25.2–52.4)	36.2 (22.9–49.9)
10 mg/kg, treatment naïve (*N *=* *21)	65.9 (45.0–85.7)	57.7 (33.3–81.0)	57.7 (33.3–81.0)	49.5 (23.8–75.4)
10 mg/kg, previously treated (*N *=* *37)	49.9 (33.3–66.6)	31.6 (16.5–47.6)	28.4 (13.9–44.2)	28.4 (13.9–44.2)

CI, confidence interval; NR, not reported.

1 Based on Kaplan–Meier estimation with CIs computed using the bootstrap method; analyses include all randomized patients for Weber et al. 27 and Wolchok et al. 26, and all treated patients for O'Day et al. 24.

2 CI not available for Hodi et al. 16 and Robert et al. 23.

3 In the 0.3 and 3 mg/kg dose groups, 33% and 42% of patients, respectively, crossed over to the 10 mg/kg dose group.

A second phase III trial reported by Robert et al. treated patients at a higher dose of 10 mg/kg, included 502 patients with treatment naïve metastatic melanoma, and confirmed the long-term survival benefit associated with ipilimumab (Table1) 23. In this study, OS was significantly longer for ipilimumab in combination with dacarbazine compared with dacarbazine plus placebo (11.2 months vs. 9.1 months). The OS rates at 1, 2, and 3 years also demonstrated a significant benefit for patients receiving ipilimumab; for patients in the ipilimumab plus dacarbazine arm, 47.3%, 28.5%, and 20.8%, respectively; and for placebo plus dacarbazine, 36.3%, 17.9%, and 12.2%, respectively (HR: 0.72; *P *<* *0.001) 23.

Schadendorf et al. reported an analysis of 1861 patients treated with ipilimumab in 12 prospective and retrospective trials for which survival data were available. This study reported a median OS of 11.4 months (95% CI: 10.7–12.1). This report also included 254 patients with 3-year follow-up data. Three-year OS rates were 22% for the entire population, 26% for treatment naïve patients, and 20% for previously treated patients. The OS curve reached a plateau at year 3 independent of whether patients were treatment naïve or had received maintenance therapy. Among 4846 patients, the median OS was 9.5 months (95% CI: 9.0–10.0) with a plateau in the OS curve beginning around year 3 for 21% of the patients 28. Table1 summarizes the survival rates of different patient populations treated in multiple phase II studies 25. Patients enrolled in the three phase II studies reported by O'Day et al. 24, Wolchok et al. 26, and Weber et al. 27 have demonstrated 4-year OS rates between 13.8% and 49.5%. Altogether, these data demonstrate that ipilimumab provides durable responses and a survival benefit to a subset of patients with metastatic melanoma.

## Patterns of Response

Due to its immune modulating mechanism of action, ipilimumab produces a range of response patterns that in many cases differ from the responses traditionally observed in patients treated with conventional cytotoxic agents 29. Similar to cytotoxic therapy, following treatment with ipilimumab, patients may experience a rapid decline of baseline tumor lesions and no evidence of new lesions. Other groups of patients may experience stable disease, which in some cases can be followed by ongoing slow and steady decline of tumor burden 29. Two additional response patterns observed throughout the clinical development of ipilimumab are novel and are also associated with positive patient outcomes. A group of patients may experience initial increase in their tumor burden followed by complete disappearance of all tumors. This type of response is believed secondary to infiltrating T-cell conglomerates around the tumors giving the radiographic appearance of greater tumor size and hence, progressive disease. Positron emission tomography computed tomography (PET-CT) scans are not able to differentiate due to the high fluorodeoxyglucose uptake in both inflammation and neoplastic disease. In some cases, patients will also have a reduction in the total tumor burden during or after the appearance of new lesions that may ultimately regress; which may be due to the fact that the activated immune system may require time to mount an effective response 29.

It is very important to stress that regardless of the clinical or radiographic response, for the most part, patients who derive the benefits of CTLA-4 inhibition experience an improvement in their clinical performance status and organ function (such as liver function and serum lactic dehydrogenase) profiles. Both the traditional and the new response patterns are associated with favorable survival 29. In response to these initial observations, investigators participating in anti-CTLA-4 clinical trials developed new response criteria for the evaluation of immune therapy; thus, the terminology of immune-related response criteria (irRC) has emerged. While not fully validated, the irRC are currently being prospectively evaluated for a broader use in trials evaluating immunotherapeutic agents 18.

These novel response patterns emphasize the need for a confirmation of progressive disease prior to moving on to additional therapeutic options for a patient. Follow-up scans should be completed to confirm true tumor progression; this is particularly important in patients who appear to be stable or improving after therapy. This clinical judgment is critical to avoid early termination of a treatment that may benefit patients with limited therapeutic options.

## Management of Toxicities

In keeping with the immune stimulatory mechanism of ipilimumab, it is not surprising that the safety profile for this agent includes inflammatory, immune-mediated side effects which may resemble or differ from the side effects observed after therapy with cytotoxic agents developed to date. A recent analysis of 14 pooled ipilimumab clinical trials has evaluated the overall safety profile of the agent 30. All patients in the studies included in the retrospective analyses had unresectable stage III or stage IV melanoma and no prior history or clinical evidence of autoimmune disease or treatment with immunosuppressive drugs. Patient characteristics, such as age, prior treatment history, and performance, status varied among trials, which were conducted at various doses of ipilimumab ranging from 0.1 to 20 mg/kg. Importantly, the safety events included in this analysis were only those reported between the first dose and 70 days after the last dose of study therapy 30.

**Figure 1:**
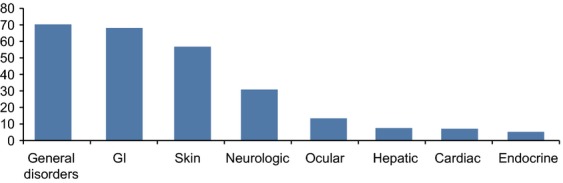
Percentage of any grade adverse events (AEs) 30. In a retrospective review of 1498 patients using safety data from 14 completed clinical trials, AEs were categorized by organ system. AEs were included regardless of causality. Patients may have experienced more than one event.

Almost all patients in this analysis experienced an AE, with an incidence of 96.9% for any grade AE (46.9% grade 3/4) and an incidence of 84.8% for any grade drug-related AE (25.3% grade 3/4). The most common AEs included those affecting the gastrointestinal (GI) tract (i.e., diarrhea, nausea, abdominal pain) and skin (i.e., rash, pruritus) (Fig.1). The majority of AEs appeared to be related to the agent's mechanism of action and, therefore, are categorized as immune-related AEs (irAEs). Most irAEs are of low-grade severity, but are part of a severity spectrum, and, as such, may progress to a more severe grade. Based on the experience during clinical development, there is a potential for severe complications (i.e., intestinal perforation/colectomy) with fatal outcome. Similar to AEs reported regardless of causality, the most common irAEs affect the skin (44.9%) and GI tract (32.5%). Close supervision and prompt recognition of collateral irAEs may lead to early treatment with corticosteroids and control of the symptoms in a majority of patients. Nonetheless, death may still occur in less than 1% of patients 30.

This analysis also confirmed the overall safety profile observed in individual, randomized phase III trials and did not find new safety concerns 16,23,30. Median time to onset for any irAE grades 2–4 is 6.86 weeks (95% CI: 4.14–8.43) after initiation of treatment, but occasionally they can occur weeks or months after the last dose of ipilimumab 16,30,31. Management guidelines have been developed through the clinical trial development process for ipilimumab and tremelimumab (Table2) 32,33 and strongly advise initiation of corticosteroids in any patient treated with ipilimumab in which an irAE event is suspected. The duration and intensity of the corticosteroid intervention and the decision of discontinuation of ipilimumab are based on the severity of the toxicity observed 30,32,34. The Common Terminology Criteria for Adverse Events (CTCAE) provides clinical descriptions of severity for AEs based on the general guideline where grade 1 represents a mild AE; grade 2, moderate; grade 3, severe; grade 4, life-threatening or disabling AE; and grade 5, death related to AE. Table3 provides CTCAE definitions for the common irAEs (involving the GI tract and skin) observed with ipilimumab 35.

**Table 2:** Guidelines for recommended management of irAEs 32,33

Site	Signs and symptoms	Management
GI	Assess patients for changes in bowel habits and for the following signs and symptoms: diarrhea, abdominal pain, blood or mucus in stool with or without fever, peritoneal signs consistent with bowel perforation, and ileus	Low-grade events: symptomatic management (dietary modifications and loperamide) High-grade events: corticosteroid therapy may be required >7 stools/day over baseline, signs consistent with perforation, or patients with a fever: administer 1–2 mg/kg prednisone or equivalent and then move forward with ensuring differential diagnosis Withhold ipilimumab for moderate reactions until improvement to mild severity or complete resolution; for severe reactions, discontinue ipilimumab
Skin	Evaluate patients for signs and symptoms of pruritus, vitiligo, or maculopapular rash	Mild to moderate: symptomatic management. Topical moisturizers and oatmeal baths may help relieve mild casesModerate to severe: topical and/or systemic corticosteroids may be requiredWithhold ipilimumab dosing in patients with moderate to severe signs and symptomsPermanently discontinue ipilimumab in patients with Stevens–Johnson syndrome, toxic epidermal necrolysis, or rash complicated by full thickness dermal ulceration or necrotic, bullous, or hemorrhagic manifestations
Liver	Run liver function tests (LFTs) before each infusion or more frequently if possibleMonitor patients for any signs of hepatitis	Moderate AST or ALT >2.5 times but ≤5 times ULN, or moderate total bilirubin elevation >1.5 times but ≤3 times ULN: withhold ipilimumab dose Severe AST or ALT elevations of >5 times ULN; total bilirubin elevations of >3 times ULN; or failure to complete full treatment course within 16 weeks from administration of first dose: permanently discontinue ipilimumab Grade ≥3 hepatitis: consider corticosteroid therapy
Endocrine	Nonspecific symptoms include: fatigue, headache, changes in mental status, abdominal pain, unusual bowel habits, and hypotensionUndertake appropriate blood work	Moderate reactions or symptomatic endocrinopathy: withhold ipilimumab until complete resolution or stable on hormone replacement therapyPatients unable to have their corticosteroid dose reduced to 7.5 mg prednisone or equivalent per day: permanently discontinue ipilimumab Consider long-term hormone replacement therapy as necessary
Neurologic	Encourage patient report of changes in muscle weakness or sensory alternations	New onset or worsening symptoms: may require permanent discontinuation of ipilimumab
Ocular	Assess patients for uveitis, iritis, or episcleritis	Administer corticosteroid drops

ALT, alanine aminotransferase; AST, aspartate aminotransferase; LFTs, liver function tests; ULN, upper limit of normal.

**Table 3:** Common terminology criteria for adverse events: grading and definition of rash and GI events 35

AE	Grade 1	Grade 2	Grade 3	Grade 4	Grade 5
Rash	Macular or papulareruption or erythemawithout associatedsymptoms	Macular or papulareruption or erythema with pruritus or otherassociated symptoms; localized desquamation or other lesions covering <50% of BSA	Severe, generalized erythroderma or macular, papular or vesicular eruption; desquamation covering ≥50% BSA	Generalized exfoliative, ulcerative, or bullous dermatitis	Death
Pruritus	Mild or localized	Intense or widespread	Intense or widespread and interfering with ADL	–	–
Diarrhea	Increase of <4 stools per day over baseline; mild increase in ostomy output compared to baseline	Increase of 4–6 stools per day over baseline; IV fluids indicated <24 h; moderate increase in ostomy output compared to baseline; not interfering with ADL	Increase of ≥7 stools per day over baseline; incontinence; IV fluids ≥24 h; hospitalization; severe increase in ostomy output compared to baseline; interfering with ADL	Life-threatening consequences (e.g., hemodynamic collapse)	Death
Nausea	Loss of appetite without alteration in eating habits	Oral intake decreased without significant weight loss, dehydration or malnutrition; IV fluids indicated <24 h	Inadequate oral caloric or fluid intake; IV fluids, tube feedings, or TPN indicated ≥24 h	Life-threatening consequences	Death
Abdominal pain	Mild pain not interfering with function	Moderate pain; pain or analgesics interfering with function, but not interfering with ADL	Severe pain; pain or analgesics severely interfering with ADL	Disabling	–

ADL, activities of daily living; BSA, body surface area; TPN, total parenteral nutrition.

## Future Directions

Despite the large knowledge base accumulated about ipilimumab and its mechanism of action, several challenges regarding its safety and potential benefits remain. Ongoing and future studies will help to optimize dosing, management of toxicities, and applications to patients with melanoma and other malignancies. Many of these answers will be obtained by astute observation of patients treated on a daily basis 36, whereas others will come from the results of ongoing and planned postmarketing clinical trials aimed at improving our understanding of the science associated with ipilimumab's novel mechanism of action.

The ultimate dosing of ipilimumab has not been fully established. The dose of 3 mg/kg administered every 3 weeks for a maximum of four infusions was initially validated in a 3-arm phase III study comparing ipilimumab with gp100 and a combination of both agents among 676 patients with advanced melanoma 16. More recent data studying the efficacy of ipilimumab administered at 10 mg/kg in combination with dacarbazine compared with dacarbazine and placebo also demonstrated a survival benefit with a similar toxicity profile. A dose-finding study reported by Wolchok et al. compared three different dose levels and demonstrated a significantly superior overall response rate among patients treated at 10 mg/kg (11.1%) compared with those treated at 3 mg/kg (4.2%) and 0.3 mg/kg (0%) 26. An ongoing phase III study will compare 3 mg/kg versus 10 mg/kg ipilimumab 37.

Another intriguing and challenging concept is that of an “individualized” dosing based on the ability of ipilimumab to break the host's peripheral immune tolerance and recognize antigen epitopes on the surface of melanoma cells. This concept is based on the notion of individual thresholds of antigen recognition. An attempt to elucidate this concept was made by Maker et al. The investigators treated 46 patients with metastatic melanoma to explore whether intrapatient dose escalation would induce greater autoimmunity and antitumor activity. Escalating doses of the antibody resulted in proportionally higher plasma concentrations. Sixteen patients (35%) experienced grade III/IV irAEs. Five patients (11%) achieved an objective clinical response. The study explored three dose levels: 3, 5, and 9 mg/kg. Antitumor activity was evaluated using Response Evaluation Criteria in Solid Tumors (RECIST). Hypophysitis (*n* = 8/19), and diarrhea (*n* = 6/19) were the predominant grade 3/4 irAEs 38. While a major effort to test this concept, this clinical trial took place early in the development of ipilimumab and the doses explored were below 10 mg/kg. Nonetheless, it is important to note that the ORR observed at this dose level is identical to that of subsequent trials at 10 mg/kg and the incidence of irAEs is in keeping with the doses explored to date. Higher dose levels for patients who do not develop irAEs may be associated with greater responses and still an interesting concept to further study.

### Re-exposure to ipilimumab

Other aspects deserving further study are those of maintenance and retreatment 39. A retrospective analysis by Caroline Robert and others looked at the duration of responses among 40 of the 676 patients (6%) who experienced clinical benefit from therapy with ipilimumab in the phase III trial comparing the antibody with gp100, a peptide vaccine and were retreated with a regimen consisting of four doses of ipilimumab. Patients who had experienced greater than grade 3 skin toxicities or grade 4 toxicities (except for endocrinopathies controlled with appropriate hormonal therapy) were excluded. Theoretically, this concept addresses the very basic concepts of tumor heterogeneity, immune-editing, and antigenic immunogenicity. In this retrospective analysis, the authors demonstrated a greater duration of responses experienced by approximately 13% of patients retreated with the agent in the pivotal phase III study. Acknowledging the limited number of patients in this analysis, it is important to note that: (1) none of the patients with unresectable stage III were retreated; (2) seven of the 40 patients retreated attained a better response after retreatment than with the original regimen, and (3) a third of the patients in this cohort had already experienced progression of disease, and re-exposure to the agent induced further antitumor activity in about 19% (*n* = 6/31) of them. Hence, re-exposure or retreatment with ipilimumab (previously known as reinduction) may be considered for patients who do not experience severe toxicities and develop progression of disease after an initial response, or beyond 3 months of disease stabilization as currently recommended by the National Comprehensive Cancer Center Network guidelines 40.

Improving the benefits of ipilimumab is also a challenge for the coming years. While strategies combining CTLA-4 blockade and vaccination remain of intellectual interest, the data available with different vaccine strategies are limited and difficult to interpret. This may be due to the limited number of patients studied, or in the case of gp100, the lack of an overall additional benefit from the vaccines developed to date 16,41. Other trials of great interest explore the safety and efficacy of ipilimumab in combination with other immune modulators, and targeted therapies including anti-VEGF and EGFR monoclonal antibodies, and BRAF inhibitors in melanoma and other solid tumors; combinations with chemotherapy in lung, prostate, melanoma, pancreas, and other tumor types. However, initial experience demonstrates the need for caution when evaluating combinations because of the potential for greater toxicity. Data from a trial evaluating the concurrent combination of ipilimumab and vemurafenib at their currently approved doses, or with a lower dose of vemurafenib, in patients with metastatic melanoma resulted in greater hepatotoxicity than expected for either agent alone 42.

### Combinations with radiation therapy

The combination of ipilimumab with radiation therapy gained major attention during the last 5 years. While several clinical observations suggest the potential induction of an abscopal effect with this approach, and a preliminary publication further generated enthusiasm 36, its ultimate additional benefit has not been clearly confirmed. This concept has been studied mainly in patients with central nervous system (CNS) involvement where the combination is more frequently applied. However, this setting may not be the ideal one due to obvious pharmacodynamic considerations and a prospective clinical trial is in progress. A retrospective analysis of 58 patients with melanoma and limited brain metastases, who were treated with stereotactic radiosurgery at a median dose of 20 Gy, included 25 patients treated with concomitant IV ipilimumab at 3 mg/kg every 3 weeks for a median of four doses. The study demonstrated that the cause of death was CNS progression in 50 patients (86%). There was no statistical difference in local control, lack of new brain lesions, intracranial hemorrhage, or OS between either group 43. In contrast, a cohort of 77 patients with melanoma and brain involvement in which 35% of the patients received ipilimumab was reported by Knisely et al. and differed substantially in its results 44. The median survival of patients receiving ipilimumab was 21.3 months, as compared with 4.9 months among those who did not. Another retrospective report by Silk et al. further supported an improved OS among 70 patients with melanoma metastatic to the brain 45. Of these, 33 patients received ipilimumab and 37 did not. The patients who received ipilimumab experienced a median survival of 18.3 months (95% CI: 8.1–25.5), compared with 5.3 months (95% CI: 4.0–7.6) for those patients who did not receive ipilimumab. Barker et al. from Memorial Sloan-Kettering Cancer Center retrospectively analyzed 29 patients who underwent 33 courses of nonbrain radiotherapy between their first and last dose of ipilimumab encountered that the toxicity profile is similar and that the administration of both treatments does not affect the palliative effects of radiation nor the survival benefit of ipilimumab therapy 46. Ongoing prospective studies will shed further lights on this aspect of therapy.

### Activity in patients with brain involvement

In addition to the retrospective analyses mentioned above, Margolin et al. conducted a phase II trial looking at this particular issue. Seventy-two patients with melanoma and brain metastases were enrolled in two different cohorts: cohort A included neurologically asymptomatic patients who were not receiving steroid therapy at study entry; and cohort B included symptomatic patients treated with a stable dose of corticosteroids. All patients received four doses of IV ipilimumab at 10 mg/kg every 3 weeks. Patients who were clinically stable after 6 months were eligible to receive 10 mg/kg IV ipilimumab every 12 weeks. After 12 weeks, nine of 51 (17.6%) of patients in cohort A experienced disease control, compared with 1 out of 21 (4.7%) patient in cohort B (5%, 0.1–24). Brain control was achieved in 12 of 51 (24%) patients in cohort A and 2 of 21 (10%) patients in cohort B. There was disease control outside of the brain in 14 of 51 (27%) patients in cohort A and in 1 of 21 (5%) patient in cohort B. Toxicities were similar to other ipilimumab trials. These data suggest that ipilimumab is active in some patients with brain involvement, in particular if the metastatic deposits are small and asymptomatic 47.

### Combinations with other immune checkpoint modulators

The discovery of CTLA-4 and its clinical applications for the treatment of human malignancies led to more intense research initiatives exploring other immune checkpoints. Another member of this family of receptors is the programmed death 1 (PD-1) protein which is a coinhibitory receptor expressed on B cells and activated or exhausted T cells. It has a similar structure to CTLA-4 but different biological function and specificity for ligands. PD-1 has two known ligands: PD-L1 (B7-H1) and PD-L2 (B7-DC). Greater affinity for PD-L1 has been recognized. PD-L1 is selectively expressed and inducible in lymphoid, and nonlymphoid tissues; in different tumors 48 and in other cells of the tumoral microenvironment, in response to inflammatory stimuli 49. PD-L1 expression is associated with worse outcome in different tumor types 50,51. Additionally, it is also evident that the PD-1/PD-L1 pathway can be used by tumoral cells for their own protection from immunological responses mediated by T cells 52,53.

A wealth of clinical and important basic science has been generated during the last decade involving inhibitors of the PD-1 pathway. While Bristol-Myers Squibb is developing a fully human monoclonal antibody against PD-1, known as nivolumab (MDX 1106, BMS-936558, Bristol-Myers Squibb), Merck Inc. is developing pembrolizumab (MK3475, lambrolizumab; Merck Inc., Whitehouse Station, NJ), an IgG4 humanized monoclonal antibody against PD-1. Similarly, PD-L1 has been targeted by EMD Serono with MSB0010718C (EMD Serono, Inc., a subsidiary of Merck KGaA, Darmstadt, Germany), MEDI4736 (Astra Zeneca, London, England), and Genentech is studying a MPDL3280A (RG7446; Genentech, South San Francisco, CA). While the antitumor activity of these compounds is rapidly being studied and confirmed in melanoma and other malignancies, nivolumab and pembrolizumab have also demonstrated durable responses in patients with melanoma. As expected, the observed toxicities are mainly immune in nature and similar to those resulting from CTLA-4 inhibition 54.

The survival benefit of nivolumab in melanoma was reported by Topalian et al. in a prospective analysis of 107 patients with advanced melanoma treated with the antibody every 2 weeks at different dose levels. The median OS was 16.8 months, and the 1- and 2-year survival rates reached 62% and 43%, respectively. For 33 patients who experienced objective tumor responses (31%), the estimated median duration of response was 2 years. In addition to confirming durable antitumor activity, the investigators in this study also noted no additional toxicities 55. Similarly, the antitumor efficacy of pembrolizumab has been reported by Hamid et al. 56 and Robert et al. 57. More recently, Ribas et al. reported on the durable nature of these responses among 411 patients with advanced disease. Of them, 221 patients had prior exposure to ipilimumab, and 190 were ipilimumab-naïve. The investigators studied three different dose schedules: 162 patients were treated with 2 mg/kg every 3 weeks, 192 patients received 10 mg/kg every 3 weeks, and 57 patients received the 10 mg/kg dose every 2 weeks. The ORR was 28% for patients with previous ipilimumab therapy, and 40% for patients with no prior ipilimumab therapy. The estimated 1-year survival rate was 69% (74% in ipilimumab-naïve patients and 65% for those previously treated); the median PFS was 24 weeks for ipilimumab-naïve patients and 23 weeks for the previously treated patients 58.

Recent data have demonstrated an additional benefit when ipilimumab is administered in combination with anti-PD-1 agents. In the first study exploring this combination regimen, Wolchok et al. reported on 86 patients with melanoma treated with ipilimumab every 3 weeks for 4 doses, followed by nivolumab alone every 3 weeks for 4 doses (concurrent regimen) 59. Thirty-three patients received nivolumab every 2 weeks for up to 48 doses (sequential regimen). Objective responses were observed in 40% of patients who received the concurrent regimen in comparison with only 20% when the agents were administered sequentially. At the maximum selected doses (nivolumab at a dose of 1 mg/kg of body weight and ipilimumab at a dose of 3 mg/kg) 53% of the patients experienced objective responses. Additionally, about one-half of the patients experienced grade 3 or 4 treatment-related AEs which were, for the most part, reversible and similar to those observed with either antibody alone. This initial experience has generated major enthusiasm among investigators and patients, and at least 10 clinical trials are exploring the ideal dosing, safety, and ultimate efficacy of this combination regimen.

## Conclusions

The safety and efficacy of CTLA-4 blockade with ipilimumab has been evaluated in a number of phase I, II, and III trials demonstrating that therapy with ipilimumab may result in a significant improvement in OS for a selected group of patients with metastatic melanoma. The survival benefit for patients treated in different clinical trials now exceeds 4 years in some cases 25. Most patients (84.8%) experience a drug-related AE of any grade, and most of these are immune related of low toxicity grade, according to the US National Cancer Institute CTCAE. However, a minority of patients may also experience severe and life-threatening AEs. In clinical studies, AEs were managed according to guidelines that emphasized close clinical monitoring and early use of corticosteroids when appropriate. Despite greater understanding in the biology, pharmacokinetics, safety, and efficacy of ipilimumab, several aspects of its full development remain to be investigated. At present, more than 150 clinical trials continue to explore these aspects comparing dose levels alone or in combination with other immune-mediated therapies, establishing pharmacodynamics, safety, and efficacy when administered in combination with chemotherapeutic agents, tyrosine kinase inhibitors, angiogenesis inhibitors, and agents with selective affinity against PD-1 and PD-L1. The data generated by these trials will ultimately find their position in cancer therapy over the next decade. Finally, the lessons learned with CTLA-4-blocking antibodies have already proved critical to our understanding and further development of other immune checkpoint inhibitors.

## References

[b1] Siegel R, Ma J, Zou Z, Jemal A (2014). Cancer statistics, 2014. CA Cancer J. Clin.

[b2] Jemal A, Devesa SS, Hartge P, Tucker MA (2001). Recent trends in cutaneous melanoma incidence among whites in the United States. J. Natl Cancer Inst.

[b3] Korn EL, Liu PY, Lee SJ, Chapman JA, Niedzwiecki D, Suman VJ (2008). Meta-analysis of phase II cooperative group trials in metastatic stage IV melanoma to determine progression-free and overall survival benchmarks for future phase II trials. J. Clin. Oncol.

[b4] Davies H, Bignell GR, Cox C, Stephens P, Edkins S, Clegg S (2002). Mutations of the BRAF gene in human cancer. Nature.

[b5] Chapman PB, Hauschild A, Robert C, Haanen JB, Ascierto P, Larkin J (2011). Improved survival with vemurafenib in melanoma with BRAF V600E mutation. N. Engl. J. Med.

[b6] Hauschild A, Grob JJ, Demidov LV, Jouary T, Gutzmer R, Millward M (2012). Dabrafenib in BRAF-mutated metastatic melanoma: a multicentre, open-label, phase 3 randomised controlled trial. Lancet.

[b7] Flaherty KT, Robert C, Hersey P, Nathan P, Garbe C, Milhem M (2012). Improved survival with MEK inhibition in BRAF-mutated melanoma. N. Engl. J. Med.

[b8] Flaherty KT, Infante JR, Daud A, Gonzalez R, Kefford RF, Sosman J (2012). Combined BRAF and MEK inhibition in melanoma with BRAF V600 mutations. N. Engl. J. Med.

[b9] Cheng S, Chu P, Hinshaw M, Smith K, Maize J, Sferruzza A (2011). Frequency of mutations associated with targeted therapy in malignant melanoma patients. J. Clin. Oncol.

[b10] Brunet JF, Denizot F, Luciani MF, Roux-Dosseto M, Suzan M, Mattei MG (1987). A new member of the immunoglobulin superfamily–CTLA-4. Nature.

[b11] Brunet JF, Denizot F, Golstein P (1988). A differential molecular biology search for genes preferentially expressed in functional T lymphocytes: the CTLA genes. Immunol. Rev.

[b12] Krummel MF, Allison JP (1995). CD28 and CTLA-4 have opposing effects on the response of T cells to stimulation. J. Exp. Med.

[b13] Ribas A, Camacho LH, Lopez-Berestein G, Pavlov D, Bulanhagui CA, Millham R (2005). Antitumor activity in melanoma and anti-self responses in a phase I trial with the anti-cytotoxic T lymphocyte-associated antigen 4 monoclonal antibody CP-675,206. J. Clin. Oncol.

[b14] Camacho LH, Antonia S, Sosman J, Kirkwood JM, Gajewski TF, Redman B (2009). Phase I/II trial of tremelimumab in patients with metastatic melanoma. J. Clin. Oncol.

[b15] Ribas A, Kefford R, Marshall MA, Punt CJ, Haanen JB, Marmol M (2013). Phase III randomized clinical trial comparing tremelimumab with standard-of-care chemotherapy in patients with advanced melanoma. J. Clin. Oncol.

[b16] Hodi FS, O'Day SJ, McDermott DF, Weber RW, Sosman JA, Haanen JB (2010). Improved survival with ipilimumab in patients with metastatic melanoma. N. Engl. J. Med.

[b17] Melero I, Hervas-Stubbs S, Glennie M, Pardoll DM, Chen L (2007). Immunostimulatory monoclonal antibodies for cancer therapy. Nat. Rev. Cancer.

[b18] Hoos A, Ibrahim R, Korman A, Abdallah K, Berman D, Shahabi V (2010). Development of ipilimumab: contribution to a new paradigm for cancer immunotherapy. Semin. Oncol.

[b19] Weber J (2010). Immune checkpoint proteins: a new therapeutic paradigm for cancer—preclinical background: CTLA-4 and PD-1 blockade. Semin. Oncol.

[b20] Boasberg P, Hamid O, O'Day S (2010). Ipilimumab: unleashing the power of the immune system through CTLA-4 blockade. Semin. Oncol.

[b21] Camacho LH (2008). Novel therapies targeting the immune system: CTLA4 blockade with tremelimumab (CP-675,206), a fully human monoclonal antibody. Expert Opin. Investig. Drugs.

[b22] Young SE, Giuliano AE, Morton DL (2005). Three decades of evolving treatment for melanoma: no improvement in survival?. J. Clin. Oncol.

[b23] Robert C, Thomas L, Bondarenko I, O'Day S, Weber J, Garbe C (2011). Ipilimumab plus dacarbazine for previously untreated metastatic melanoma. N. Engl. J. Med.

[b24] O'Day SJ, Maio M, Chiarion-Sileni V, Gajewski TF, Pehamberger H, Bondarenko IN (2010). Efficacy and safety of ipilimumab monotherapy in patients with pretreated advanced melanoma: a multicenter single-arm phase II study. Ann. Oncol.

[b25] Wolchok JD, Weber JS, Maio M, Neyns B, Harmankaya K, Chin K (2013). Four-year survival rates for patients with metastatic melanoma who received ipilimumab in phase II clinical trials. Ann. Oncol.

[b26] Wolchok JD, Neyns B, Linette G, Negrier S, Lutzky J, Thomas L (2010). Ipilimumab monotherapy in patients with pretreated advanced melanoma: a randomised, double-blind, multicentre, phase 2, dose-ranging study. Lancet Oncol.

[b27] Weber J, Thompson JA, Hamid O, Minor D, Amin A, Ron I (2009). A randomized, double-blind, placebo-controlled, phase II study comparing the tolerability and efficacy of ipilimumab administered with or without prophylactic budesonide in patients with unresectable stage III or IV melanoma. Clin. Cancer Res.

[b28] Schadendorf D, Hodi FS, Robert C, Weber JS, Margolin K, Hamid O (2013). Pooled analysis of long-term survival data from phase II and phase III trials of ipilimumab in metastatic or locally advanced, unresectable melanoma. Eur. J. Cancer.

[b29] Wolchok JD, Hoos A, O'Day S, Weber JS, Hamid O, Lebbé C (2009). Guidelines for the evaluation of immune therapy in solid tumors: immune-related response criteria. Clin. Cancer Res.

[b30] Ibrahim R, Berman D, de Pril V, Humphrey RW, Chen T, Messina M (2011). Ipilimumab safety profile: summary of findings from completed trials in advanced melanoma. J. Clin. Oncol.

[b31] Dummer R MMaio, Hamid O, O'Day SJ, Richards J, Wolchok JD (2010).

[b32] http://www.yervoy.com/hcp/rems.aspx.

[b33] Shaw SA, Camacho LH, McCutcheon IE, Waguespack SG (2007). Transient hypophysitis after cytotoxic T lymphocyte-associated antigen 4 (CTLA4) blockade. J. Clin. Endocrinol. Metab.

[b34] Squibb B-M (2011). YERVOY (ipilimumab) [prescribing information].

[b35] Cancer Therapy Evaluation Program (CTEP) http://ctep.cancer.gov.

[b36] Postow MA, Callahan MK, Barker CA, Yamada Y, Yuan J, Kitano S (2012). Immunologic correlates of the abscopal effect in a patient with melanoma. N. Engl. J. Med.

[b37] ClinicalTrials.gov (2012). http://www.clinicaltrials.gov/ct2/show/NCT01515189.

[b38] Maker AV, Yang JC, Sherry RM, Topalian SL, Kammula US, Royal RE (2006). Intrapatient dose escalation of anti-CTLA-4 antibody in patients with metastatic melanoma. J. Immunother.

[b39] Robert C, Schadendorf D, Messina M, Hodi FS, O'Day S, MDX investigators (2013). Efficacy and safety of retreatment with ipilimumab in patients with pretreated advanced melanoma who progressed after initially achieving disease control. Clin. Cancer Res.

[b40] http://www.nccn.org/professionals/physician_gls/PDF/melanoma.pdf.

[b41] Ribas A, Comin-Anduix B, Chmielowski B, Jalil J, de la Rocha P, McCannel TA (2009). Dendritic cell vaccination combined with CTLA4 blockade in patients with metastatic melanoma. Clin. Cancer Res.

[b42] Ribas A, Hodi FS, Callahan M, Konto C, Wolchok J (2013). Hepatotoxicity with combination of vemurafenib and ipilimumab. N. Engl. J. Med.

[b43] Mathew M, Tam M, Ott PA, Pavlick AC, Rush SC, Donahue BR (2013). Ipilimumab in melanoma with limited brain metastases treated with stereotactic radiosurgery. Melanoma Res.

[b44] Knisely JP, Yu JB, Flanigan J, Sznol M, Kluger HM, Chiang VL (2012). Radiosurgery for melanoma brain metastases in the ipilimumab era and the possibility of longer survival. J. Neurosurg.

[b45] Silk AW, Bassetti MF, West BT, Tsien CI, Lao CD (2013). Ipilimumab and radiation therapy for melanoma brain metastases. Cancer Med.

[b46] Barker CA, Postow MA, Khan SA, Beal K, Parhar PK, Yamada Y (2013). Concurrent radiotherapy and ipilimumab immunotherapy for patients with melanoma. Cancer Immunol. Res.

[b47] Margolin K, Ernstoff MS, Hamid O, Lawrence D, McDermott D, Puzanov I (2012). Ipilimumab in patients with melanoma and brain metastases: an open-label, phase 2 trial. Lancet Oncol.

[b48] Dong H, Strome SE, Salomao DR, Tamura H, Hirano F, Flies DB (2002). Tumor-associated B7-H1 promotes T-cell apoptosis: a potential mechanism of immune evasion. Nat. Med.

[b49] Curiel TJ, Wei S, Dong H, Alvarez X, Cheng P, Mottram P (2003). Blockade of B7-H1 improves myeloid dendritic cell-mediated antitumor immunity. Nat. Med.

[b50] Thompson RH, Kuntz SM, Leibovich BC, Dong H, Lohse CM, Webster WS (2006). Tumor B7-H1 is associated with poor prognosis in renal cell carcinoma patients with long-term follow-up. Cancer Res.

[b51] Brahmer JR, Drake CG, Wollner I, Powderly JD, Picus J, Sharfman WH (2010). Phase I study of single-agent anti-programmed death-1 (MDX-1106) in refractory solid tumors: safety, clinical activity, pharmacodynamics, and immunologic correlates. J. Clin. Oncol.

[b52] Hirano F, Kaneko K, Tamura H, Dong H, Wang S, Ichikawa M (2005). Blockade of B7-H1 and PD-1 by monoclonal antibodies potentiates cancer therapeutic immunity. Cancer Res.

[b53] Jin HT, Ahmed R, Okazaki T (2011). Role of PD-1 in regulating T-cell immunity. Curr. Top. Microbiol. Immunol.

[b54] Ganghadar TC, Vonderheide RH (2014). Mitigating the toxic effects of anticancer immunotherapy. Nat. Rev. Clin. Oncol.

[b55] Topalian SL, Sznol M, McDermott DF, Kluger HM, Carvajal RD, Sharfman WH (2014). Survival, durable tumor remission, and long-term safety in patients with advanced melanoma receiving nivolumab. J. Clin. Oncol.

[b56] Hamid O, Robert C, Daud A, Hodi FS, Hwu WJ, Kefford R (2013). Safety and tumor responses with lambrolizumab (anti-PD-1) in melanoma. N. Engl. J. Med.

[b57] Robert C, Ribas A, Wolchok JD, Hodi FS, Hamid O, Kefford R (2014). Anti-programmed-death-receptor-1 treatment with pembrolizumab in ipilimumab-refractory advanced melanoma: a randomised dose-comparison cohort of a phase 1 trial. Lancet.

[b58] Ribas A, Hodi FS, Kefford R, Hamid O, Daud A, Wolchok JD (2014). Efficacy and safety of the anti-PD-1 monoclonal antibody MK-3475 in 411 patients (pts) with melanoma (MEL). J. Clin. Oncol.

[b59] Wolchok JD, Kluger H, Callahan MK, Postow MA, Rizvi NA, Lesokhin AM (2013). Nivolumab plus ipilimumab in advanced melanoma. N. Engl. J. Med.

